# Global Implementation of Tobacco Demand Reduction Measures Specified in Framework Convention on Tobacco Control

**DOI:** 10.1093/ntr/ntab216

**Published:** 2021-10-18

**Authors:** Heikki Hiilamo, Stanton Glantz

**Affiliations:** 1 National Institute for Health and Welfare, Helsinki, Finland; 2 Department of Social Research, University of Helsinki, Helsinki, Finland; 3 Department of Medicine, University of California, San Francisco, San Francisco, CA, USA

## Abstract

**Introduction:**

The world’s first global health treaty, WHO Framework Convention on Tobacco Control (FCTC) aims to reduce tobacco product demand by focusing on tobacco taxes, smoking bans, health warning labels, and tobacco advertising bans. Previous studies almost unanimously suggest that FCTC has prompted countries to implement more effective tobacco demand reduction policies.

**Aims and Methods:**

By taking into account the pre-FCTC status, country income level, and state capacity we studied if ratifying FCTC was associated with tobacco demand reduction measures in 2018/2019. We used logistic regression to assess the association of FCTC ratification with adoption demand reduction measures, accounting for years since ratification, baseline status, and other covariates.

**Results:**

Except for taxes, state of tobacco policy implementation before FCTC ratification did not predict adoption of FCTC policies. Time since FCTC ratification was associated with implementing smoking bans and pictorial HWLs. In contrast, while the tax rate prior to FCTC ratification was positively associated with increased taxes after FCTC ratification, time since FCTC ratification was marginally negatively associated with increases in tobacco taxes.

**Conclusions:**

While the FCTC was followed by implementation of compliant demand reduction policies, there are still many parties that have not implemented the FCTC, particularly increasing taxes and ending tobacco advertising and promotions.

**Implications:**

We assessed changes in tobacco demand reductions measures over 22 years in 193 countries. By using internal tobacco industry documents, we were able establish a baseline before the FCTC negotiations. Unlike previous studies, we included four tobacco demand reductions measures: tobacco taxes, smoking bans, health warning labels, and tobacco advertising ban. The limitation of the study is that we do not have data to describe if demand reduction measures are actually enforced or what their effect on tobacco consumption is.

## Introduction

The WHO Framework Convention on Tobacco Control (FCTC) is the first global health treaty negotiated under the auspices of the World Health Organization.^1^ After the World Health Assembly adopted the treaty in 2003 and it entered into force in 2005, it became one of the most rapidly and widely embraced treaties in United Nations history with 181 parties by July 2020.^2^ The articles on tobacco product demand reduction measures include Article 6 (price and tax measures), Article 8 (protection from exposure to tobacco smoke), Article 11 (packaging and labeling of tobacco products, including health warning labels, HWL) and Article 13 (tobacco advertising, promotion and sponsorship; TAPS). The FCTC conference of parties (COP) has adopted specific guidelines to implement each of these articles.^3–6^ Despite progress in implementing these policies, the success in implementing FCTC compliant demand reduction policies remains incomplete.^5,7–11^ This study complements qualitative data from in-country visits on the role of the FCTC^12^ with a quantitative assessment of the association of FCTC ratification with national implementation of FCTC tobacco demand reduction measures.

The tobacco industry has fought to undo or to weaken tobacco demand reduction measures. The industry has opposed tax increases (Article 6) by commissioning research claiming economic benefits of tobacco, creating alliances, including with both progressive and conservative organizations, lobbying ministries of finance with poor knowledge of public health and FCTC requirements, and arguing tax increases drive illicit trade and hurt disadvantaged groups.^13–23^ They have also learnt how to cope with tax increases and sometimes actually benefit from them by over-shifting taxes on premium brands to increase profits while downshifting taxes on ultra-low-price brands to cushion the effects of tax increases on total consumption.^22,24,25^ The tobacco industry has collaborated with (and financed) the hospitality^19^ and gambling industries^26^ to maintain smoking in public places (Article 8), particularly presenting ventilation as an alternative to smoke-free environments.^20,27^

The industry opposes effective health warning labels (HWL, Article 11) through submissions to government, privately influencing politicians, and the media, using third parties to argue the industry’s position, commissioning research (including opinion polls and legal research) arguing that people already know the hazards of smoking, arguing that HWLs conflict with other national laws, including copyright and trademarks, and international trade treaties and through litigation.^15,18,23,28^

The tobacco industry has promoted voluntary agreements over legislation and sought loopholes to continue promoting tobacco products as alternatives to tobacco advertising, promotion, and sponsorship bans (Article 13).^7,29^

Research papers (Supplementary Table S1) and official FCTC progress reports^30,31^ demonstrate that FCTC has prompted countries to implement more effective tobacco demand reduction policies.^6,32^ Provisions of the FCTC that set targets for the speed of implementation of health warning labels and TAPS restrictions (Articles 11 [HWL^8,9,33]^ and 13 [TAPS]^7^) are the most implemented^32^ while taxation measures lag behind.^11,34^ The FCTC accelerated the adoption of smoke-free policies,^10,33^ but they remained the least implemented measure among 22 Eastern Mediterranean countries.^35,36^

The final objective of demand reduction measures is to curb the use of tobacco products. However, there are conflicting results concerning the association between FCTC implementation and reduction in cigarette smoking. Three papers^33,37,38^ show positive association between FCTC implementation and reduction in smoking prevalence. In contrast, another^39^ found no significant change in the rate at which global cigarette consumption had been decreasing after the FCTC’s adoption in 2003, using either interrupted time series analysis or event modeling. The results were robust to the year FCTC negotiations commenced (1999) and the year when the FCTC first became legally binding in each country. However, the authors of this paper did not consider the time lag from ratification to policy change.

There are at least four challenges to quantitatively evaluating the effects of FCTC on tobacco control policies which may also explain conflicting results of FCTC outcomes. First, countries may have had FCTC-compliant policies already in place before FCTC entered into force, which means that the FCTC would not have affected the policy. Second, if the countries were not compliant with FCTC requirements before the process towards FCTC started in 1999 they may have had some restriction in place at that time. In this case, a progress in FCTC implementation could mirror path-dependency where the countries that started with less stringent demand reduction policies would have progressed towards FCTC requirements even without the treaty or, alternatively, the existence of less stringent policies could have blocked further progress. Third, the success in implementing demand reduction policies may have to do with income level of countries. Fourth, implementation of demand reduction measures may depend on state capacity. With rising awareness of health consequences of smoking countries that have a higher state capacity could have implemented more effective tobacco demand reduction policies also in the absence of FCTC.^7–11^

The aim of this study is to quantitatively analyze FCTC success by taking a two-decade-long perspective and analyzing the development of tobacco demand reduction measures accounting for pre-FCTC policies in 1997 eight years before, and in 2019, 14 years after the treaty first entered force in individual countries, which accounts for the fact that countries ratified the FCTC at different times. This study also complements previous analysis by extending the time frame of the analysis and by allowing comparison across the four areas of demand reduction measures.

## Research Design and Variables

### Independent Variables

We studied the effect of the FCTC on demand reduction laws by calculating the number of years since FCTC ratification by each country as of 2019. The assumption is that implementing FCTC is a cumulative process over time. Countries that had signed but not ratified the FCTC as of the end of 2019 (Argentina, Cuba, Haiti, Morocco, Switzerland, and USA), or had not signed or become parties to the FCTC by the end of 2019 (Andorra, Dominican Republic, Eritrea, Indonesia, Malawi, Monaco, Saint Kitts and Nevis, Saint Lucia, and Somalia) had years since ratification set to 0. We did not include Taiwan, which the United Nations considers being represented by the People’s Republic of China. The dataset had 193 countries.

We addressed the problem of pre-FCTC status by establishing a pre-FCTC baseline from internal tobacco industry documents to see if the countries were FCTC compliant before the end of 1990s. The industry documents available at Truth Tobacco Industry Documents (https://www.industrydocuments.ucsf.edu/tobacco/) are the only comprehensive source on tobacco control implementation dating back beyond FCTC ratification. We identified the relevant documents through keyword searches (including smoke-free policy, health warning, health warning label, tax policy).^9^ We also used the industry documents to determine if the countries had any demand reduction restrictions which were not FCTC-compliant but could indicate that the countries had already started to implement some tobacco control measures in the particular area or that they had adopted industry-friendly policies as an alternative to meaningful public health policies. The hypothesis is that it would be easier for countries with some tobacco control policies in 1997 to later implement FCTC compliant policies. Alternatively, countries that had adopted weak policies could be “stuck” at that level of policy development. Either situation would suggest a path-dependency towards FCTC compliance in 2019.

We addressed the issue with income level by World Bank 2019 gross national income (GNI) categories. According to a July 2019 report released by World Bank, low-income economies were defined as those with a GNI per capita US$1025 or less in 2017 (coded 1); lower middle-income economies are those with a GNI per capita between $1026 and $3,995 (coded 2); upper middle-income economies are those with a GNI per capita between $3,996 and $12,375 (coded 3); high-income economies are those with a GNI per capita of $12,376 or more (coded 4).^40^ We did not consider any changes occurring in income levels between 1997 and 2019.

We quantified the state capacity (or, more precisely, incapacity) with The Fund for Peace’s fragile state’s index as of 2019 for 178 countries.^41^ The Fragile States Index is based on 12 indicators covering a wide range of state failure risk elements such as extensive corruption and criminal behavior, inability to collect taxes or otherwise draw on citizen support, large-scale involuntary dislocation of the population, sharp economic decline, group-based inequality, institutionalized persecution or discrimination, severe demographic pressures, brain drain, and environmental decay. In 2019 Yemen had the highest score (113.5) while Finland had the lowest score (16.9). State fragility is supposedly closely associated with a state’s capacity to implement public policies including tobacco control measures.^8^

### Dependent Variables

The outcome we used for each FCTC tobacco demand reduction measure (Article 6: price and tax measures, Article 8: protection from exposure to tobacco smoke, Article 11: packaging and labeling of tobacco products, Article 13: TAPS) was compliance with the FCTC as described in the following paragraphs.

### Tobacco Tax Policies

FCTC Article 6 commits parties to implement “tax policies and, where appropriate, price policies, on tobacco products so as to contribute to the health objectives aimed at reducing tobacco consumption.” Article 6 implementation guidelines recommend tax policies that consider tobacco products’ price elasticity (the rate by which tobacco consumption decreases as result of price increases) and income elasticity (the sensitivity of tobacco consumption to income changes) to make tobacco products less affordable over time.^4^ However, neither Article 6 nor the guidelines set specific targets for taxes or prices.

Tobacco tax rate is the portion of the price represented by all taxes, including value-added tax (VAT) for the most-sold brand of cigarettes. Since FCTC Article 6 does not set any specific tax rate we used an outcome variable derived from MPOWER standards in the WHO Report on the Global Tobacco Epidemic 2015: Raising Taxes on Tobacco.^42^ The highest standard is taxes that totaled at least 75% of retail price. The expectation in MPOWER is that if the manufacturers increase wholesale prices so that the overall tax rate drops below 75%, the government would increase taxes so that the tax share would go above 75%.

Data on the tobacco tax rate, including specific excise, ad valorem excise, import duties, VAT, and other taxes were obtained from the WHO Report on the Global Tobacco Epidemic 2019 public dataset.^32^ This dataset includes information collected by WHO experts as of July 2018 on the prices of the most-sold brand of cigarettes (both in local currency and in US$) and cigarette taxes.^32^ In countries where different taxes applied to cigarettes based on length of cigarette, quantity produced, or type (e.g. filter vs. non-filter), the rate that applied to the most-sold brand was used.

Unlike for three policy areas mentioned below we were not able to find comprehensive pre-FCTC data for the year 1997. Instead, we obtained baseline pre-FCTC taxes using the 1999 World Bank survey of 64 countries that reported the share of cigarette taxes (including VAT) as a percentage of the retail price of a pack of cigarettes^43^ supplemented by the tobacco industry’s International Tobacco Documentation Centre’s 1998 International Fiscal Guide to Tobacco^44^ that mapped international taxation, price, and tariff policies. Both data sources include information on retail price and tax for the most-sold cigarette brand. The high correlation (0.947, *p* < .001) for overlapping price information for the 46 countries which were included in both datasets indicates the data have been collected in a substantially uniform manner (57 countries were not in both datasets). The correlation for tax data was lower (0.676, *p* < .001). This lower correlation, while still significant, could indicate a measurement error in the datasets or it could indicate variability in tobacco taxes and cigarette prices increases from 1998 to 1999. Given the more reliable international standing, we deemed the WB survey more reliable for those countries which were included in both datasets than the International Fiscal Guide to Tobacco produced by the tobacco industry. The dataset to study tax policies had 102 countries. To study path-dependency we studied the effect of having a 50% tobacco tax rate in 1999, the second-highest MPOWER standard, on having a 75% tax rate in 2018.

### Smoke-Free Policies

FCTC Article 8 commits countries to “adopt and implement measures, providing for protection from exposure to tobacco smoke in indoor workplaces, public transport, indoor public places and, as appropriate, other public places.” Parties have five years after the convention enters into force for that party to meet the requirements. This means that if a country ratified the treaty in 2010, it committed itself to implement Article 8 by 2015.

We obtained baseline data on national smoking restriction laws from the tobacco industry–created International Tobacco Documentation Centre’s Smoking Issues Status Book for 1997^45^ (workplaces, cafes and restaurants, and bars and nightclubs) and from the WHO report on the global tobacco epidemic in 2019,^32^ which gives the status of smoke-free environments as of 31 December 2018 (healthcare facilities, educational facilities except universities, universities, government facilities, indoor offices, restaurants, pubs, and bars).^32^

Both datasets included eight indicators for smoking bans: health care facilities, educational facilities, universities, governmental facilities, indoor offices, restaurants, pubs, and the public transportation systems) for each country. For the WHO report, we classified as smoke-free all the cases marked in the reports as “yes” being smoke-free, meaning that the law mandated complete smoke-free spaces, and classified the cases listed as “no” in the reports as not smoke-free. We scored missing data as no restrictions. For the Smoking Issues Status Book we coded venues for each countries as ban (0,1) when a ban was mentioned and restrictions (0,1) when restrictions (any other restriction mentioned besides a ban) were mentioned. For analysis, we chose those two venues which had lowest level of bans in 1997: pubs (no bans in 1997) and indoor offices (13 countries with bans). Missing data were coded as no restriction (19 countries for pubs and 12 countries for indoor offices in 2019).

### Health Warnings on Cigarette Packs

FCTC Article 11 specifies that HWLs shall be approved by the competent national authority, shall be rotating, shall be large, clear, visible, and legible, should be 50% or more of the principal display areas but shall be no less than 30% of the principal display areas, and maybe in the form of or include pictures or pictograms.^1^ Parties have three years after the convention enters into force to meet the requirements. As outcome variable int 2019, we used those countries with HWLs covering at least 50% on average of the principal display areas and which include a photograph or graphic.

To establish a baseline for HWLs in 1997 we used previous work which analyzed the FCTC compliance of HWL in 1997 on the basis of data collected from internal tobacco industry documents.^28^ Compliant countries had HWL’s which were at least 30% of the principal display areas. In 1997 no country has HWLs with pictures or pictograms. We scored missing data as no mandated HWLs (*n* = 26). Missing data were coded as no restriction (33 countries in 2019).

### Tobacco Advertising, Promotion and Sponsorship (TAPS) Bans

Article 13 requires prohibition of TAPS “that promote a tobacco product by any means that are false, misleading or deceptive or likely to create an erroneous impression about its characteristics, health effects, hazards or emissions”. In absence of complete bans, Article 13 requires “that health or other appropriate warnings or messages accompany all tobacco advertising and, as appropriate, promotion, and sponsorship; and restrict the use of direct or indirect incentives that encourage the purchase of tobacco products by the public.” Article 13 guidelines^46^ emphasize that “a ban on tobacco advertising, promotion, and sponsorship is effective only if it has a broad scope.” Parties have five years after the convention enters into force for that party to meet the requirements.

To establish a pre-FCTC baseline we used information collected by the tobacco industry’s International Tobacco Documentation Centre’s (a tobacco industry organization that monitored policy for the companies^47^) for its 1997 Smoking Issues Status Book (SISB) that listed advertising and promotion regulations in 179 countries or autonomous regions for direct (TV, radio, movies, press, billboards, and point-of-sale) and indirect (sponsorship, whether tobacco brand or company names are permissible on non-tobacco products) advertising.^47^ To create categories corresponding to WHO data, we collapsed bans on national TV and radio (96 countries had TV bans and 87 countries had radio bans in 1997); information on TAPS bans for national press, billboards, point-of-sale, sponsorship, and brand advertising were used as reported. For analysis, we chose those two areas, which had lowest level of bans in 1997: sponsorship (21 countries with bans in 1997) and point-of-sale advertising (23 countries with bans). There were no missing data for 2019.

### Statistical Analysis

By using cross-sectional data from 2019 we examined associations between FCTC compliance (dependent variable) with logistic regression model, where the independent variables were years since the countries had ratified FCTC, pre-existing measures (restrictions) in 1997 (variable for path-dependency), country income group (factor variable where low-income countries were the reference category) and state capacity. We report results from the full model where all variables were included. We used statistical software R 4.03.

### Patient and Public Involvement

Patients or the public were not involved in the design, or conduct, or reporting, or dissemination plans of our research.

## Results

At baseline in 1997, there were no countries with smoking bans in pubs or countries having health warning labels with a graphic element (Table 1). The highest compliance rate was for 75% tobacco tax rate (13%, 13 countries). In 2018/2018 the highest compliance rate was observed for graphic health warning labels (58%) while 75% tobacco tax rate had the lowest compliance rate of 20% (39 countries).

**Table 1.** Share of FCTC Compliant Countries at Two Time Points

Demand reduction measure	1997/1999 (before FCTC)	2018/2019 (after FCTC)
75% tobacco tax rate	13%	20%
Smoking ban in pubs	0%	40%
Smoking ban in indoor offices	7%	45%
Graphic health warning labels	0%	58%
Tobacco sponsorship ban	11%	30%
Ban on point-of-sale advertising	12%	51%

*N* = 193, except for tax policies where *n* = 102.

The logistic regression analysis showed that time since FCTC ratification was statistically significant for passing smoking bans in pubs and indoor offices (OR 1.13 [1.03–1.27], per year since ratification, *p* = .015; 1.13 [1.03–1.26] per year since ratification, *p* = .013) and FCTC compliant HWL’s in with pictures or pictograms (OR 1.17 [1.06–1.31] per year since ratification, *p* = .002,) (Table 2). There was also a suggestion that FCTC ratification was associated with sponsorship and point-of-sales advertising bans (OR 1.10 [0.99–1.23] per year since ratification, *p* = 0.090; 1.08 [0.99–1.19] per year since ratification, *p* = .096). Conversely, we found a suggestion that time since FCTC ratification had a negative association with having 75% tobacco tax rate in 2018 (OR 0.89 [0.78–1.01] per year, *p* = 0.069).

**Table 2.** Logistic Regression Results for Four Areas of Tobacco Demand Reduction Measures

	Taxes (2018)		Smoking bans (2019): pubs		Smoking bans (2019): indoor offices		Health warning labels (2019)		TAPS (2019): sponsorship		TAPS (2019): point-of-sale	
	Estimate [95% CI]	p	Estimate [95% CI]	p	Estimate [95% CI]	p	Estimate [95% CI]	p	Estimate [95% CI]	p	Estimate [95% CI]	p
Time since FCTC ratification	*0.89* *[0.78–1.01]*	*.069*	**1.13 [1.03–1.27]**	**.015**	**1.13 [1.03–1.26]**	**.013**	**1.17 [1.06–1.31]**	**.002**	*1.10 [0.99–1.23]*	*.090*	*1.08 [0.99–1.19]*	*.096*
50% tax rate (1999)	**3.53** **[1.06–13.10]**	**.047**										
Smoking restrictions (1997)			1.35 [0.47–3.87]	.576	1.04 [0.50–2.14]	.924						
FCTC compliant HWLs (1997)							5.32 [0.86–103.9]	.132				
TAP restrictions (1997)									1.15 [0.47–2.74]	0.755	0.92 [0.42–2.02]	.835
WB income group												
1 Low-income	Ref		Ref		Ref		Ref		Ref		Ref	
2 Lower middle-income	0.88 [0.08–20.93]	.919	0.48 [0.16–1.41]	.185	1.17 [0.40–3.41]	.774	**3.75 [1.16–13.70]**	**.033**	**0.29 [0.09–0.89]**	**.034**	0.92 [0.30–2.79]	.883
3 Higher middle- income	5.38 [0.46–144.04]	.221	1.40 [0.45–4.46]	.561	1.57 [0.47–5.30]	.460	*3.14 [0.83–13.07]*	*.099*	0.35 [0.10–1.24]	.107	0.66 [0.19–2.28]	.510
4 High-income	2.53 [0.08–130.31]	.641	0.56 [0.10–2.94]	.498	0.64 [0.11–3.48]	.608	2.46 [0.38–16.98)	.349	0.29 [0.04–1.81]	.194	2.36 [0.39–15.09]	.353
State capacity	1.01 [0.96–1.06]	.803	1.00 [0.98–1.03]	.991	1.00 [0.97–1.02]	.714	**0.96 [0.93–0.99]**	**.018**	0.99 [0.96–1.02]	.451	*1.02 [1.00–1.05]*	*.071*

Statistically significant estimates (p<0.05) are presented in bold. Almost statistically significant estimates (*p* < 0.10) are italicized.

Income group mattered for HWL’s and tobacco sponsorship bans where lower middle-income countries were more likely to have passed graphic HWLs (3.75 [1.16–13.70], *p* = .033) but less likely to have passed sponsorship bans (OR 0.29 [0.09–0.89], *p* = .034) than low-income countries. Countries with lower state capacity were slower in passing HWL (OR 0.96 [0.93–0.99], *p* = .018) than countries with higher state capacity. We found that countries with at least 50% tobacco tax rate in 1999 more often had a 75% tax rate in 2018 (OR 3.53 [1.06–13.10], *p* = .047).

## Discussion

This quantitative study complements qualitative data from in-country visits on the role of the FCTC^12^ to identify four challenges to evaluating the effects of FCTC implementation: countries may have had FCTC-compliant policies already in place before FCTC entered into force, if the countries were not compliant with FCTC requirements they may have had some restriction in place at that time, the success in implementing demand reduction policies may have to do with income level of countries, and finally implementation of demand reduction measures may depend on state capacity. Our results showed the only pre-FCTC policy associated with implementation was having at least 50% tobacco tax rate in 1997 was associated with having at least 75% tobacco tax rate in 2018. The lower middle-income countries had higher likelihood of passing HWLs but lower likelihood of passing sponsorship bans. Unlike in earlier analysis apart from a single weak indication (HWL’s) we did not find consistent evidence for state capacity to play a role in FCTC demand reduction measure outcomes.

Aligning with earlier research,^6,48^ we found that time since FCTC ratification is positively associated with passing of smoking bans (indoor offices and pubs) and HWLs. Our results align with an earlier analysis showing that FCTC ratification was not associated with passing point-of-sales and sponsorship ban, the two area of TAPS bans with lowest level of implementation in 1997.^7^ However, also in these venues there was weak indication of positive association with FCTC ratification

Our results highlight the low implementation rate in tax policies, which are an effective measure in reducing cigarette consumption and smoking-related diseases.^42^ Indeed, we found a marginally negative association between time since FCTC ratification and implementation of 75% tobacco tax rate. It is possible that countries ratifying FCTC have neglected weakly defined Article 6 measures while concentrating on implementing other demand reduction measures which are more clearly defined in the treaty. A complementary explanation is that FCTC ratifying countries had 50% or higher (but below 75%) tobacco tax rates already before FCTC process. The health ministries are usually responsible for other FCTC demand reduction articles, while the tobacco tax policies fall usually under finance ministries, which often have closer contacts with tobacco industry.

Income group was not consistently associated with implementation of tobacco demand reduction measures, which may indicate that the determined efforts from tobacco control community with substantial financial support by international charities for low-income countries to participate in international effort to implement FCTC compliant tobacco control measures^6^ might be yielding positive results. Participation in Conference of Parties (COP) was correlated with stronger tobacco control policies in low-income countries.^49^ Also, the MPOWER program introduced by WHO in 2008 with funding from The Bill & Melinda Gates Foundation and The Bloomberg Philanthropies has been effective in promoting tobacco demand reduction policies.^34–36,50^

Our results question the positive reports of FCTC impact on tobacco tax policies.^5,6,33,51–53^ The FCTC Global progress report 2018 demonstrates high compliance rates for demand reduction measures than WHO data (Table 2). In Article 6 (price and tax measures) rate was 64% among 181 signatories, 88% in Article 8 (protection from exposure to tobacco smoke), 77% in Article 11 packaging and labeling of tobacco products) and 61% in Article 13 (TAPS).^30^ The results from this study especially the figure for Article 6 appears inflated. The observations in the FCTC Global progress report database are contingent on FCTC parties submitting their implementation reports and answering all questions on the relevant indicators in those reports, while the data used in this study is based on WHO’s Global Report on the Tobacco Epidemic. These data are obtained and validated independently by WHO through its regional and country offices and do not rely on countries’ commitment to fill out the reporting instrument. Previous research has also discovered discrepancies in parties’ implementation reports.^52^ There is an urgent need to coordinate and possibly combine WHO Global Report data collection and FCTC Global progress report data collection across different categories of tobacco products.

### Limitations

This analysis focused on how much a country is actually putting relevant laws and regulations into effect. Some of the FCTC effects might not be visible in our data since passing FCTC compliant legislation package may take several years. We do not have data to describe if legislated demand reduction measures are actually enforced or what their effect on tobacco consumption is. We coded initial policy status using tobacco industry documents; it had been desirable to cross-verify these policies using other sources, but were not able to find relevant comparative data sources other than tobacco tax rates. The WHO data for 2019 included some missing values for pubs, indoor offices, and health warning labels. As a sensitivity analysis, we excluded these countries. The results remained basically unchanged. We did not study the passing of other tobacco demand reduction policies or tobacco supply reduction policies. We did not have uniform data sources for baseline.

## Conclusion

As time passed after FCTC ratification countries were more likely implement smoking bans and HWLs covering at least 50% on average of the principal display areas with a photograph or graphic. While tobacco taxes pre-FCTC were associated with increased taxes post-FCTC, time since FCTC ratification may have been negatively associated with increase in tobacco tax rates. Any reliable estimate of FCTC impact on implementation needs to consider the implementation status before FCTC. While the FCTC was followed by implementation of compliant demand reduction policies, there are still many parties that have not implemented the FCTC, particularly increasing taxes and ending tobacco advertising and promotions.

## Supplementary Material

A Contributorship Form detailing each author’s specific involvement with this content, as well as any supplementary data, are available online at https://academic.oup.com/ntr.

ntab216_suppl_Supplementary_Table_1

ntab216_suppl_Supplementary_Taxonomy_Form

## Data Availability

No data are shared since we are using data which are already made public.

## Patient and Public Involvement Statement

Patients or the public were not involved in the design, or conduct, or reporting, or dissemination plans of our research.
