# Supplementary material for: Global Implementation of Tobacco Demand Reduction Measures Specified in Framework Convention on Tobacco Control
Source: Nicotine Tob Res. 2021 Oct 18;24(4):503–10. doi: 10.1093/ntr/ntab216 (PMC8887591; doi:10.1093/ntr/ntab216)
Supplement: ntab216_suppl_Supplementary_Table_1 [file ntab216_suppl_supplementary_table_1.docx]

| Table 1s. Previous literature on FCTC demand reduction measures implementation outcomes | | | | |
| --- | --- | --- | --- | --- |
| Study | Research questions | Countries | Time frame | Results |
| Tumwein 2011(57) | To describe the status of tobacco control legislation in Africa in protection from exposure to tobacco smoke, packaging and labelling of tobacco products, and tobacco advertising, promotion and sponsorship. | 46 | 2011 | Despite their efforts, African countries’ FCTC implementation at national level has not matched the strong regional commitment demonstrated during the FCTC treaty negotiations. |
| Sanders-Jackson et al. 2013(9) | Pattern and passage rate of HWLs, including the effect of FCTC, and HWLs voluntarily implemented by tobacco companies. | 165 | 1965-2012 | Passage of HWLs is accelerating, and the FCTC is associated with further acceleration. Industry voluntary HWLs slowed mandated HWLs. |
| Hiilamo & Glantz 2015(8) | Effects of ratifying the FCTC, state capacity, path-dependency and tobacco industry activity on the implementation of effective health warning labels (HWL) among low -and middle-income countries. | 118 | 1992-2013 | FCTC promoted HWL policies among LMICs. The countries with voluntary HWLs in 1992 were less likely comply with FCTC 21 years later. |
| Uang et al. 2016(10) | Effect of ratifying FCTC on countries enacting smoke-free laws covering indoor workplaces, restaurants, and bars. | 166 | 1997-2012 | FCTC accelerated the adoption of smoke-free indoor workplace, restaurant, and bar laws, with the greatest effect in the years immediately following ratification. |
| Hiilamo & Glantz 2016(7) | Changes in countries’ enacting advertising bans after the effect of ratifying the FCTC. | 91-146 | 1997-2015 | Passage of complete advertising bans accelerated after FCTC ratification. Strongest development among lower middle-income countries. Lack of state capacity associated with slower progress. |
| Hiilamo & Glantz 2018(11) | Quantify changes in tobacco tax rates and cigarette affordability after countries ratified FCTC. | 103 | 1998-2014 | Implementing higher tobacco taxes remains incomplete. Ratifying FCTC not associated with higher tax rates (above 50% or above 75% of cigarette pack price). |
| Craig et al. 2019(53) | FCTC’s causal role in the implementation of tobacco control measures | 12 countries from 6 WHO regions | 2003-2015 | Tobacco industry interference continues to be a significant obstacle to further advancement. |
| Chung-Hall et al. 2019(35) | Implementation of 17 FCTC Articles during the treaty’s first decade. | All 180 parties of the convention | 2003-2016 | Implementation highest for smoke-free laws and health warnings. Price/tax increases, comprehensive smoking and marketing bans, health warnings, and cessation treatment associated with decreased tobacco consumption. |
| Valente et al. 2019(58) | Network influences on implementation of Articles 6, 8, 11,13 and 14. | All WHO countries | 2003-2016 | Diffusion effects more prevalent for pricing and taxation as well as restrictions on smoking in public places and packaging and labeling. |
| Zaatari & Bazzi 2019(59) | Impact of the WHO FCTC on non-cigarette tobacco products. | All 180 parties of the convention | 2011-2017 | The FCTC provided a framework that applies to all forms of tobacco and this encouraged some parties to adopt control measures against NCTP. Stronger global surveillance of NCTP use, tracking of policy implementation and evaluation of policy impact would assist parties in fully implementing the FCTC. |
| El-Awa et al. 2020(36) | To assess the status of MPOWER implementation in the Eastern Mediterranean Region. | 22 Eastern Mediterranean countries | 2007-2018 | 63.7% of people are covered at the highest level for the smoke-free policies measure; 60.7% for the health warnings measure; 29,4% for the advertising bans measure; and 16.1% for the taxation measure |
| Heydari 2020(38) | Trend analysis of FCTC implementation in the Eastern Mediterranean Region. | 22 Eastern Mediterranean countries | 2011-2019 | The total implementation score increased from 416 in 2011 to 509 in 2019. The lowest score increase was related to the Smoke-free Policy compliance. |
